# CitSci.org: A New Model for Managing, Documenting, and Sharing Citizen Science Data

**DOI:** 10.1371/journal.pbio.1002280

**Published:** 2015-10-22

**Authors:** Yiwei Wang, Nicole Kaplan, Greg Newman, Russell Scarpino

**Affiliations:** 1 DataONE, University of New Mexico, Albuquerque, New Mexico, United States of America; 2 Natural Resource Ecology Laboratory, Colorado State University, Fort Collins, Colorado, United States of America

## Abstract

Citizen science projects have the potential to advance science by increasing the volume and variety of data, as well as innovation. Yet this potential has not been fully realized, in part because citizen science data are typically not widely shared and reused. To address this and related challenges, we built CitSci.org (see www.citsci.org), a customizable platform that allows users to collect and generate diverse datasets. We hope that CitSci.org will ultimately increase discoverability and confidence in citizen science observations, encouraging scientists to use such data in their own scientific research.

## Abstract 2

This Community Page proposes a platform to support effective metadata documentation with a view to improving the discoverability and reusability of citizen-gathered science data.

## The Power of Citizen Science

Citizen science projects can advance science by democratizing data collection, interpretation, and analysis [1], expanding the spatial and temporal breadth of data collection [2,3] and capitalizing on the plethora and diversity of ideas generated by large numbers of people (i.e., the “wisdom of the crowd”) [4]. Given the many benefits of citizen science, it is not surprising that the number of projects, science domains involved, and volunteers engaged (both online and in person) are all increasing [5]. Despite such growth, obstacles continue to hamper discovery, understanding, and reuse of citizen science data by scientific professionals [3]. The most important obstacles involve lack of trust in data quality, transparency, and associated metadata [6]. For example, project coordinators often do not document important details regarding research questions, experimental design, and data collection and quality assurance/quality control (QA/QC) methods, which provide context about the data. Furthermore, the associated metadata do not always follow a standardized format to facilitate efficient sharing and reuse [7]. Additional barriers include a lack of tools to automate the publication of completed citizen science datasets along with the necessary metadata in existing data repositories, which, in turn, can improve data discovery and accessibility. But these obstacles are not insurmountable. We can increase confidence in (and reuse of) citizen science data by offering project coordinators improved and easy-to-use tools to document and generate associated metadata. Documentation of descriptive metadata allows people to recall the details of data collection and interpret how to use the data. Furthermore, standardization of metadata content allows for sharing of data, which may be useful or applicable to answering novel research questions [8] or in synthesis efforts, and this standardization promotes transparency in data collection efforts [9] as well as in science.

The CitSci.org support platform team is developing simple metadata tools to help project coordinators maximize the potential of citizen science data through better documentation efforts with guided prompts throughout the data life cycle [10]. Here, we describe the CitSci.org platform and discuss how we plan to improve the discoverability and reusability of citizen science data through more open and transparent documentation of data collection methods, protocols, training, and QA/QC.

## CitSci.org: A System to Bring Citizen Science Data to Life

The CitSci.org support platform (www.citsci.org) provides free and universal access for anyone to create citizen science projects. CitSci.org supports the entire data life cycle—the process from acquiring data to producing useful information—for 200+ projects (encompassing more than 407,000 measurements) by providing a centralized system for project coordinators to manage members, create custom online datasheets, explore and visualize data in real time, and download open data (from their own and other projects).

CitSci.org presently includes features supporting more rigorous sampling designs than traditional point observations, such as transects, nested-plots, and treatment replications; data quality assurance via predetermined sampling locations, species pick-lists, and acceptable data value ranges; and data validation by means of summary statistics and data exploration and visualization tools. Project coordinators can create their own measurements or use existing measurements, which are contained and searchable in an online registry across all projects. During the datasheet creation process, project coordinators are prompted to specify units of measurements for the various attributes volunteers are asked to measure. Additional features include dynamic real-time data visualizations, maps, and summary statistics and robust volunteer management features, including member communication tools, project forums, and evaluation tools. Despite this rather comprehensive suite of tools, barriers to more efficient and complete metadata documentation of citizen science data still remain. For example, specific documentation detailing original research questions, sampling designs, protocols, and methods could help scientists better appraise the value and applicability of the data available to appropriately synthesize data collected by citizen scientists for possible future reuse.

## Remaining Barriers to Citizen Science Data Reuse

While data life cycle plans should include a step that explicitly addresses data and information management, in practice, many projects fail to adequately note the details and procedures required to move from data collection planning to data reuse. For example, most references to citizen science processes generally mention disseminating results, but omit any discussion of sharing raw data for possible reuse [11]. Without proper metadata, citizen science and associated data remain suspect and, consequently, have little influence on the research proposed and conducted by professional scientists or on policy and management decisions.

Thorough documentation would improve the usefulness of citizen science data, especially if the metadata documentation were to map to domain standards adopted by specific communities, such as the DarwinCore and Ecological Metadata Language [8]. Adoption of standardized metadata practices improves data exchange possibilities and efficiencies [12] and scientific transparency, and can be used to address speculation or assumptions related to how citizen science data are collected, managed, or applied [6]. Standards for metadata enable data exchange and have mobilized citizen science data through the development of large online biodiversity data resources (e.g., the Global Biodiversity Information Facility [GBIF]), inclusion in niche modeling (e.g., State of the Birds), and applications for ongoing water quality monitoring (www.shalenetwork.org).

## Novel Approaches for Metadata Creation for Citizen Science Projects

**Fig 1:**
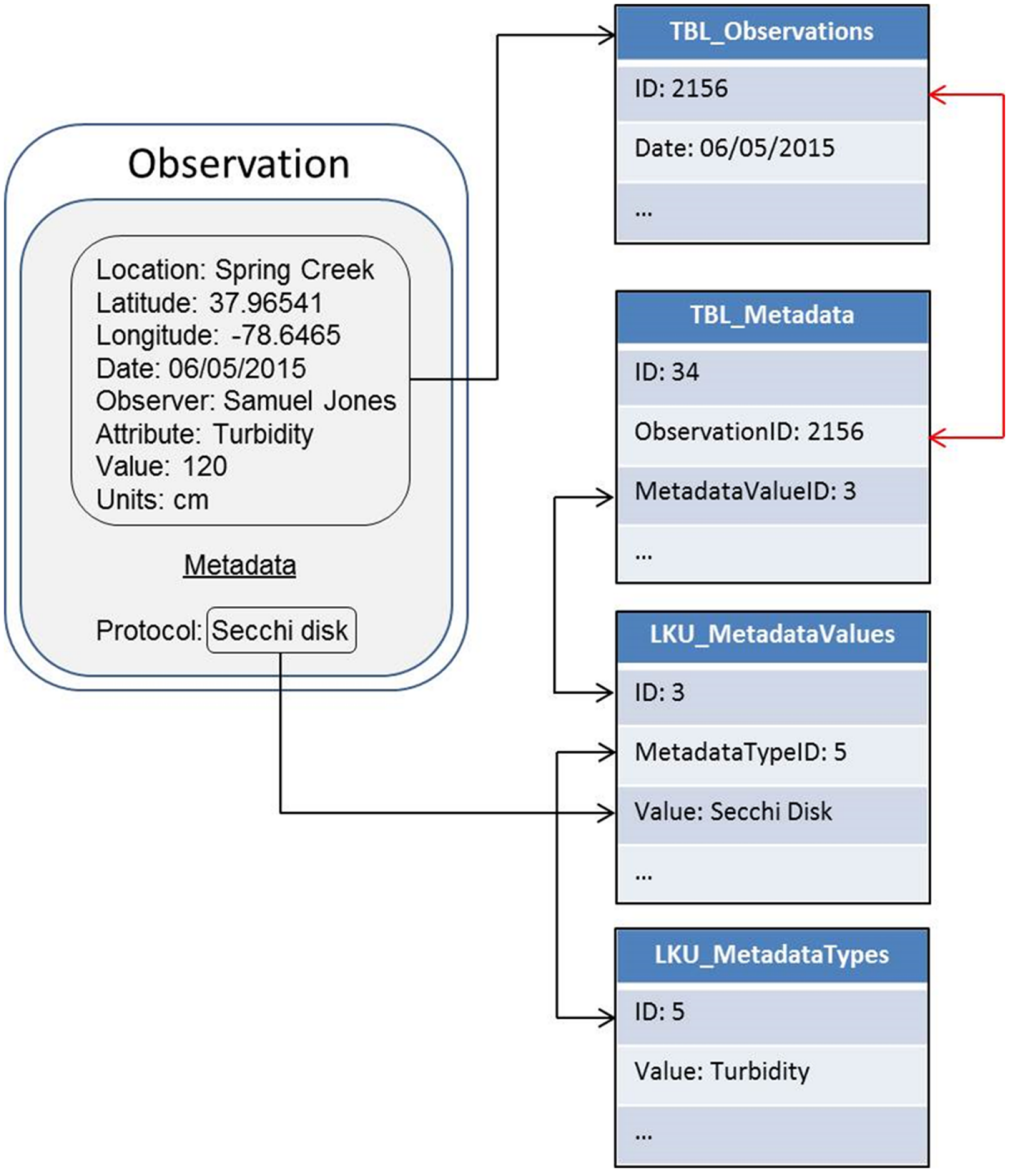
CitSci.org extensible metadata schema related to a simple field observation. An observer measures the turbidity of a stream using a Secchi disk. We capture the protocol used to measure turbidity (Secchi disk) in a metadata table (TBL_Metadata) and relate it to the observation (TBL_Observations). This extensible structure allows us to capture any type of metadata, and relate it to observations in addition to other entities, such as projects, individuals, media, etc.

To better support project coordinators’ documentation efforts, CitSci.org is building web-based data management and metadata documentation features that are flexible enough to support a variety of standards and community-driven metadata fields (Fig 1). The CitSci.org team will be developing new user interfaces that prompt project coordinators to document various aspects of their citizen science project (QA/QC, volunteer training protocols, etc.) and data collection methods (e.g., how temperature was measured) at the time of project, and online datasheet creation to produce reusable and appraisable data products. While these metadata entries will be optional, the CitSci.org platform will encourage metadata entry by displaying a progress bar and/or a similar indicator of the percentage of metadata entry complete on the project’s profile page. This approach is similar to common social media user profile completion indicators, such as is used on LinkedIn. When a project manager has entered all required project-level and data-collection–level metadata, a web badge for “Metadata Excellence Award” will appear next to the project’s photo in the project list and on the project’s profile page. Given the scalability of the CitSci.org platform’s metadata schema (Fig 1), project managers will also have the option of creating new metadata fields that may be more tailored to their specific project. All metadata fields used in CitSci.org will be displayed in a metadata registry table, viewable by all website users, and will be included in any downloads of project data. Once required metadata fields are complete, project managers can opt to share data with select data repositories (e.g., GBIF) automatically at specified intervals.

Citsci.org is developing these metadata features through a participatory approach to tailor them to citizen science practitioners’ and participants’ needs. Through a series of monthly webinars, the CitSci.org team gains input from users on specific features, with an objective of reaching consensus on how things should work. Toward that end, we encourage the scientific community to evaluate and provide feedback on the proposed metadata tools described here.

## Challenges Associated with Documenting and Sharing Citizen Science Data

Several challenges remain relating to metadata documentation and increasing transparency. To limit the initial demands on both project coordinators creating new projects and participants submitting observations online, we will adopt a reward system to encourage metadata documentation throughout the project life cycle rather than mandating it during the project creation process.

Other challenges relate to permissions, privacy, and security of the information to be documented about citizen science observations [13]. We encourage project coordinators and volunteer participants to decide which aspects of “who reported what, where, and when” to share and which to omit. Project coordinators, through recognizing the importance of proper documentation to science, also understand and respect the many concerns of the volunteers providing data and the specific nature of these data. For example, in some cases, a data provider may want to remain anonymous, while in another situation, they may want attribution. Data collected in locations that have species of concern may need to remain private or “fuzzed” to ensure precise locations cannot be identified. This raises issues related to information or data governance, which can only be resolved through close communication and co-design opportunities with project coordinators. Finally, deciding what should be documented about which facet of citizen science data can be challenging. Our approach of required metadata (the “who, what, when, where, and why”) helps focus documentation while also allowing for richer description when needed.

## A Vision for Universal Sharing of Citizen Science Data

Spurred by the advancement of internet connectivity [14], the recent growth of citizen science projects has been remarkable and promises to revolutionize and democratize science. Through collaborations with citizen scientists, professional scientists can quickly gather data in response to pressing global issues over large spatial scales and deepen connections between people and their local environment. However, issues relating to access, appraisal, trust, and use of citizen science data have so far limited its impact. By constructing tools to facilitate metadata documentation and data sharing and discovery, we aim to support greater acceptance and integration of citizen science into the broader scientific community. We expect that with increased access and transparency within citizen science, aided by easy-to-use and comprehensive, available tools, citizen science data and projects will one day fulfill their full potential and promise.
