# Correction: CitSci.org: A New Model for Managing, Documenting, and Sharing Citizen Science Data

**DOI:** 10.1371/journal.pbio.1002343

**Published:** 2015-12-18

**Authors:** Yiwei Wang, Nicole Kaplan, Greg Newman, Russell Scarpino

The Funding section incorrectly states that “the authors received no specific funding for this work.” The authors would like to correct the statement as follows:

**Funding:** YW was supported by the NSF grant 1430508. The funders had no role in study design, data collection and analysis, decision to publish, or preparation of the manuscript.
